# A diffusion tensor imaging analysis of white matter microstructures in non-operated craniosynostosis patients

**DOI:** 10.1007/s00234-022-02997-8

**Published:** 2022-06-27

**Authors:** C. A. de Planque, J. M. G. Florisson, R. C. Tasker, B. F. M. Rijken, M. L. C. van Veelen, I. M. J. Mathijssen, M. H. Lequin, M. H. G. Dremmen

**Affiliations:** 1 Department of Plastic, Reconstructive Surgery and Hand Surgery, Erasmus MC-Sophia Children’s Hospital, University Medical Center Rotterdam, Rotterdam, the Netherlands; 2 Department of Plastic, Reconstructive Surgery and Hand Surgery, Onze Lieve Vrouwe Gasthuis, Amsterdam, the Netherlands; 3 Department of Anesthesiology, Critical Care and Pain Medicine, Boston Children’s Hospital, Boston, MA USA; 4 Department of Neurosurgery, Erasmus MC-Sophia Children’s Hospital, University Medical Center Rotterdam, Rotterdam, the Netherlands; 5 Department of Radiology, UMC Utrecht, Utrecht, the Netherlands; 6 Department of Radiology and Nuclear Medicine, Erasmus MC-Sophia Children’s Hospital, University Medical Center Rotterdam, Rotterdam, the Netherlands

**Keywords:** DTI, Diffusion tensor imaging, Tractography, Craniosynostosis, Syndrome

## Abstract

**Purpose:**

In 7 to 15-year-old operated syndromic craniosynostosis patients, we have shown the presence of microstructural anomalies in brain white matter by using DTI. To learn more about the cause of these anomalies, the aim of the study is to determine diffusivity values in white matter tracts in non-operated syndromic craniosynostosis patients aged 0–2 years compared to healthy controls.

**Methods:**

DTI datasets of 51 non-operated patients with syndromic craniosynostosis with a median [IQR] age of 0.40 [0.25] years were compared with 17 control subjects with a median of 1.20 [0.85] years. Major white matter tract pathways were reconstructed with ExploreDTI from MRI brain datasets acquired on a 1.5 T MRI system. Eigenvalues of these tract data were examined, with subsequent assessment of the affected tracts. Having syndromic craniosynostosis (versus control), gender, age, frontal occipital horn ratio (FOHR), and tract volume were treated as independent variables.

**Results:**

*ʎ*_2_ and *ʎ*_3_ of the tracts genu of the corpus callosum and the hippocampal segment of the cingulum bundle show a *ƞ*^2^ > 0.14 in the comparison of patients vs controls, which indicates a large effect on radial diffusivity. Subsequent linear regressions on radial diffusivity of these tracts show that age and FOHR are significantly associated interacting factors on radial diffusivity (*p* < 0.025).

**Conclusion:**

Syndromic craniosynostosis shows not to be a significant factor influencing the major white matter tracts. Enlargement of the ventricles show to be a significant factor on radial diffusivity in the tracts corpus callosum genu and the hippocampal segment of the cingulate bundle.

**Clinical trial registration:** MEC-2014-461

**Supplementary Information:**

The online version contains supplementary material available at 10.1007/s00234-022-02997-8.

## Introduction

Patients with syndromic craniosynostosis (sCS) are at risk of developing intellectual disabilities and problems in behavioral and emotional function. Whether these derangements are caused by disturbances in brain development is unknown [1]. Mutations in genes encoding the fibroblast growth factor receptors (*FGFR*)—which are expressed during early embryonic development—are known to be responsible for the pattern of abnormal skull development in sCS [2, 3]. These gene mutations induce premature fusion of skull sutures and also affect the development of brain tissue and CSF circulation [4–6]. It is known that mutations in *FGFR-1* or *FGFR-2* are associated with decreased myelin thickness [7], but is this finding a consequence of mechanical distortion of the brain due to abnormal shape, ventriculomegaly and/or cerebellar tonsillar herniation, or does this finding reflect an intrinsic cause [8–13]?

Previously, we have reported abnormalities in brain white matter microstructure using MRI DTI in a group of operated sCS patients aged 7 to 15 years. We identified significantly higher white matter mean diffusivity (MD), axial diffusivity (AD), and radial diffusivity (RD) in the cingulate bundle, corpus callosum, and cortical spinal tract [14]. These findings suggested the presence of abnormal white matter microstructural tissue properties in sCS patients and now lead us to the purpose of our study: (1) Are these abnormalities already present in young non-operated sCS patients? (2) If so, does it reflect exposure to some mechanically related cause, like worsening ventriculomegaly, or does such an abnormality have an intrinsic cause?

We hypothesize that abnormalities in white matter architecture are already evident in non-operated children with syndromic craniosynostosis. To test our hypothesis, we used DTI-based tracts in young non-operated children with sCS.

## Material and methods

The Institution Research Ethics Board at ***, ***, approved this study (***), which is part of ongoing work at the *** Craniofacial Center involving protocolized care, brain imaging, clinical assessment, and data summary and evaluation.

### Subjects

MRIs from non-operated children with a genetically confirmed diagnosis of syndromic craniosynostosis aged under 2 years were included. The cohort consisted of patients with Apert syndrome (mutation in the fibroblast growth factor receptor (*FGFR*) 2 gene), patients with Crouzon-Pfeiffer (mutation in *FGFR* 1 or *FGFR* 2 gene), patients with Muenke syndrome (mutation in *FGFR* 3 gene), and patients with Saethre-syndrome (mutation or deletion in the twist related protein (*TWIST1* gene). Patients with 2 or more affected sutures but for whom a responsible gene mutation has not been found were named complex craniosynostosis patients. Controls with the same age range were identified from a historic hospital MRI database of children who had undergone MRI brain studies for clinical reasons between 2010 and 2020. Patients were considered a control if any cerebral and/or skull pathology was absent. Scans of potential controls were reviewed by an expert pediatric radiologist and a neurosurgeon to ensure the absence of any cerebral pathology and/or skull pathology.

### MRI acquisition

All brain MRI data were acquired with a 1.5 T unit (General Electric Healthcare, Milwaukee, Wisconsin), including three-dimensional (3D) T1-weighted fast spoiled gradient-recalled sequence, high-resolution 3D T2-weighted spin-echo sequence, and DTI sequences. DTI was obtained using a multi-repetition single-shot echo-planar sequence with a section thickness of 3 mm without a gap. Images were obtained in 25 gradient directions with the following parameters: sensitivity, b: 1000 s/mm^2^; TR: 15,000 ms; TE: 82.1 ms; FOV: 240 × 240 mm^2^; matrix: 128 × 128, resulting in a voxel size of 1.8 × 1.8 × 3.0 mm. This protocol was identical in both sCS patients and controls and kept equal throughout the entire study period.

### DTI data collection

DTI processing was performed using ExploreDTI (http://exploredti.com/). The processing consisted of correction of subject motion and eddy current distortions and a weighted linear least-squares estimation of the diffusion tensor with the robust extraction of kurtosis indices with linear estimation (REKINDLE) approach [15, 16]. White matter tracts for fiber tractography included projection fibers (corticospinal tract), commissural fibers (corpus callosum), tracts of the brain stem (medial cerebellar peduncle), and the tracts of the limbic system (fornix and cingulated bundle).

A ROI approach was used for white matter tract analysis, with the *MRI Atlas of Human White Matter* as a guideline [17]. “OR/SEED” and “AND” operators were used when tracts were allowed to pass through, and “NOT” operators were used when tracts were not allowed to pass through. Occasionally, “NOT” operators were used to avoid aberrant or crossing fibers from other bundles. To secure measuring identical parts of the different white matter tracts, 2 AND operators at both ends of a bundle to extract always the same segment of the particular white matter tract were used. We measured the tracts as reported previously [14].

### DTI metrics

The white matter metrics from DTI, voxel-by-voxel, are mathematically based on 3 mutually perpendicular eigenvectors, whose magnitude is given by 3 corresponding eigenvalues sorted in order of decreasing magnitude as *ʎ*_1_, *ʎ*_2_, and *ʎ*_3_. An ellipsoid is created by the long axis of *ʎ*_1_, and the small axes *ʎ*_2_ and *ʎ*_3,_ from where the measured length of the three axes are the eigenvalues. These eigenvalues are used to generate quantitative maps of fractional anisotropy (FA), the derivation of MD, RD, and AD. FA represents the amount of diffusional asymmetry in a voxel, which is presented from 0 (infinite isotropy) to 1 (infinite anisotropy). AD stands for the diffusivity along the neural tract: *ʎ*_1_. The diffusivity of the minor axes, *ʎ*_2_ and *ʎ*_3_, is called the perpendicular or radial diffusivity. The mean of this diffusivity *ʎ*_1_, *ʎ*_2_, and *ʎ*_3_ is known as MD. FA, MD, AD, and RD are used as indirect markers of the white matter microstructure of these young patients [18]. However, the mathematical coupling in the FA, MD, RD, and AD equations means that our statistical approach will first need to assess for differences in the eigenvalues before analyzing the impact of summary measures of diffusivity. The following equations were used:

$$\mathrm{FA}=\sqrt{\frac32}\bullet\frac{\sqrt{\left({\mathrm\lambda}_1-\mathrm{MD}\right)^2}+\left({\mathrm\lambda}_2-\mathrm{MD}\right)^2+\left({\mathrm\lambda}_3-\mathrm{MD}\right)^2}{\sqrt{\mathrm\lambda_1^2}+\mathrm\lambda_2^2+\mathrm\lambda_3^2}$$

$$\mathrm{MD}=\frac{{(\mathrm\lambda}_1+{\mathrm\lambda}_2+{\mathrm\lambda}_3)}3$$

$$\mathrm{RD}=\frac{{(\mathrm\lambda}_2+{\mathrm\lambda}_3)}2$$

$$\mathrm{AD}={\mathrm\lambda}_1$$

**Table:** 

	unit of measure
FA	scalar value ranging between 0 and 1
MD	mm^2^/s
RD	mm^2^/s
AD	mm^2^/s

### Frontal occipital horn ratio

Since ventriculomegaly is an associated abnormality in sCS patients that can affect DTI metrics, the frontal occipital horn ratio (FOHR) was used as a parameter to correct for ventricular size [19]. FOHR is defined as (frontal horn width + occipital horn width)/biparietal diameter*2 and gives a ratio of ventricle size that can be interpreted independent of age. A FOHR ≥ 0.4 was considered ventriculomegaly.

### Reliability and reproducibility

Inter-observer reliability of tract measurements was determined by comparing the results of two trained raters blinded to subject information. Both performed all structural measurements twice in 10 subjects, 5 patients, and 5 control subjects. Interrater reliability was based on 10 repeated ratings and found to be high, as depicted in Rijken et al.[14]

Partial volume effects due to brain deformity and abnormal ventricular size and shape potentially influenced the DTI fiber tractography data in patients with sCS. The FA threshold was set at 0.1 and the maximum angle threshold at 45°. This DTI fiber tractography protocol has been used in craniosynostosis patients and controls [14]. Of note, even though a FA threshold of 0.2 is commonly used [20], a threshold of 0.1 made it possible to track all structures in the control group and almost all structures in the sCS group. However, the FA threshold of 0.1 meant that more aberrant tracts were generated, and additional AND and NOT ROIs were required to exclude aberrant fibers. Additionally, by extracting particular segments from a white matter tract (by using 2 AND operators), we could measure identical white matter structures and make fair comparisons between patients with sCS and control subjects [14].

### Statistical analysis

Analyses were carried out using R Studio Version 1.1.442 – © 2009–2018 RStudio, Inc. Parametric statistics were used when the distribution of the data did not violate assumptions of normality. To minimize false positives resulting from multiple tests, multivariate analysis of variance (MANOVA) was used to determine whether patients and controls differed in patterns of *ʎ*_1_, *ʎ*_2_, and *ʎ*_3_ in the examined tracts. For *ʎ*_1_, *ʎ*_2_, and *ʎ*_3_, a *ƞ*^2^ was calculated, in which Cohen’s guideline for “high” is *ƞ*^2^ > 0.14. [21] The significant lambda values gave information from which tract FA or which diffusivity value could be affected in patients versus controls (see above, *DTI Metrics*).

Subsequent analyses used linear regression on corpus callosum genu and hippocampal segment of the left cingulate bundle with sCS/control, sex, FOHR, and tract volume added to the model as independent variables. *ß*-coefficients were calculated (stats package) for each regression. The Bonferroni correction was conducted and a *p*-value < 0.025 (*p*-value = 0.05/2) was considered statistically significant. To investigate these tracts syndromes specifically, we undertook an additional linear regression, dividing the craniosynostosis group into 5 subgroups (Apert, Crouzon, Muenke, Saethre-Chotzen, and complex). Each group was separately compared with the control group.

## Results

### Patient characteristics

**Fig. 1:**
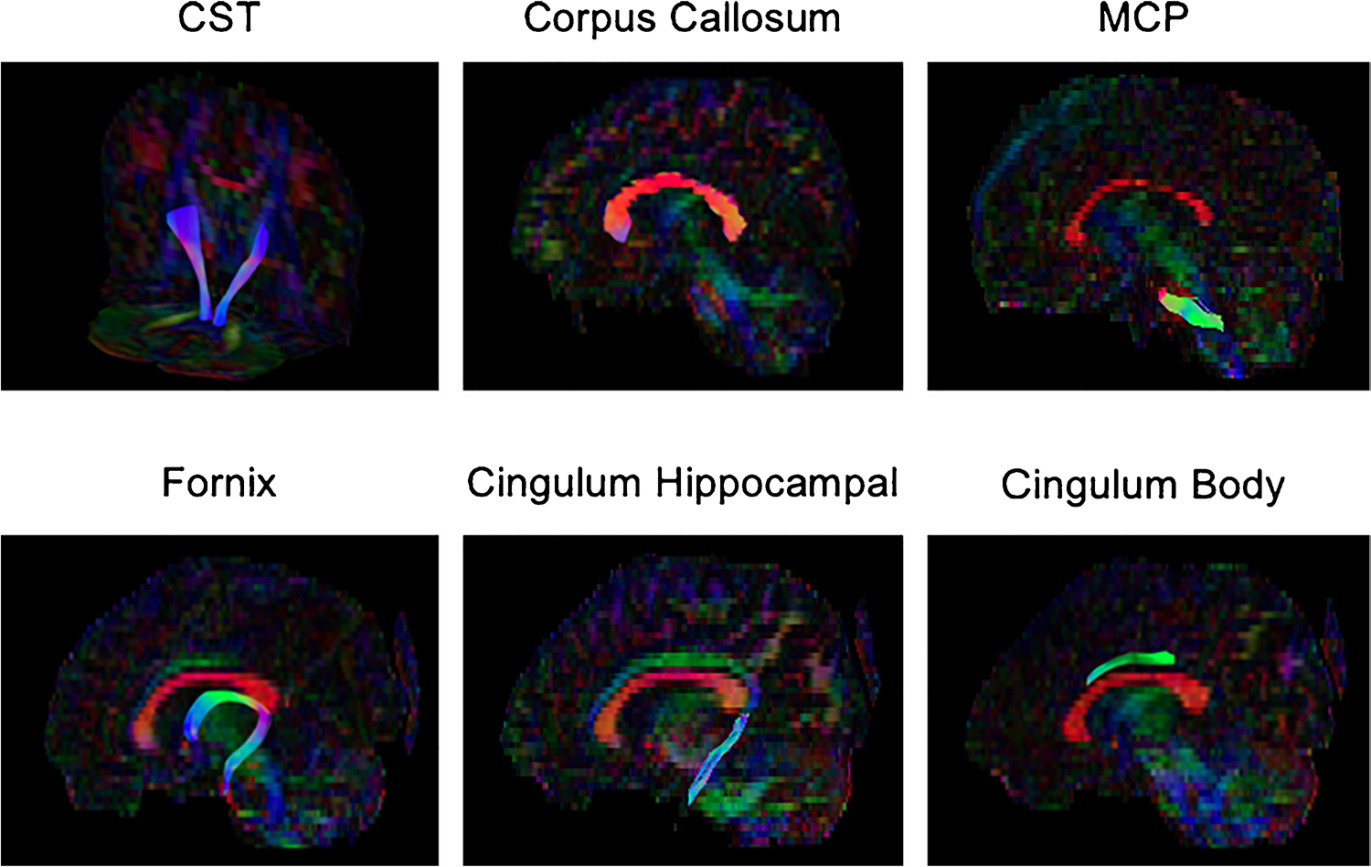
Tractography: midsegment of bilateral corticospinal tracts (CST) and midsagittal views of the corpus callosum, medial cerebellar peduncle (MCP), fornix, cingulum hippocampal segment, and cingulum body in a control patient

Fifty-one non-operated sCS patients with a median age of 0.40 [IQR 0.25] years were included, which involved Apert (*n* = 8), Crouzon-Pfeiffer (*n* = 14), Muenke (*n* = 8), and Saethre-Chotzen (*n* = 10) syndromes, and complex craniosynostosis (*n* = 11). Seventeen control subjects were included with a median age of 1.20 [IQR 0.85] years (Table 1). The measured tracts are visualized in Fig. 1.

**Table 1:** Patient characteristic

	Apert	Crouzon-Pfeiffer	Muenke	Saethre-Chotzen	Complex	Total craniosynostosis	Controls
No. of subjects M/F sex Median age (IQR)	8 4/4 0.28 (0.07)	14 5/9 0.59 (0.35)	8 1/7 0.36 (0.13)	10 5/5 0.50 (0.22)	11 2/9 0.39 (0.22)	51 17/34 0.40 (0.25)	17 5/12 1.20 (0.85)

### Eigen values ʎ_1_, ʎ_2_, and ʎ_3_

Table 2 summarizes the *ƞ*^2^ of *ʎ*_1_, *ʎ*_2_, and *ʎ*_3_ by white matter tract. The left and right hemispheres show regional asymmetries. The genu of the corpus callosum and the hippocampal segment of the left cingulum bundle show a *ƞ*^2^ > 0.14 in *ʎ*_2_ or *ʎ*_3_.

**Table 2:** Overview of *ƞ*2 of MANOVAs *ʎ*1, *ʎ*2, and *ʎ*3 in patients vs controls

	*λ*1	*λ*2	*λ*3
CST left	0.07	0.00	0.01
CST right	0.04	0.00	0.01
Corpus callosum genu	0.04	0.13	**0.14**
Corpus callosum body	0.01	0.07	0.07
Corpus callosum splenium	0.01	0.04	0.05
MCP	0.13	0.00	0.00
Fornix left	0.00	0.00	0.00
Fornix right	0.01	0.00	0.01
Cingulum hippocampal left	0.07	**0.14**	0.13
Cingulum hippocampal right	0.03	0.12	0.07
Cingulum body left	0.00	0.10	0.08
Cingulum body right	0.00	0.11	0.06

^*^For *ʎ*1, *ʎ*2, and *ʎ*3 a *ƞ*2 was calculated, in which Cohen’s guideline for “high” is *ƞ*2 > 0.14

**Fig. 2:**
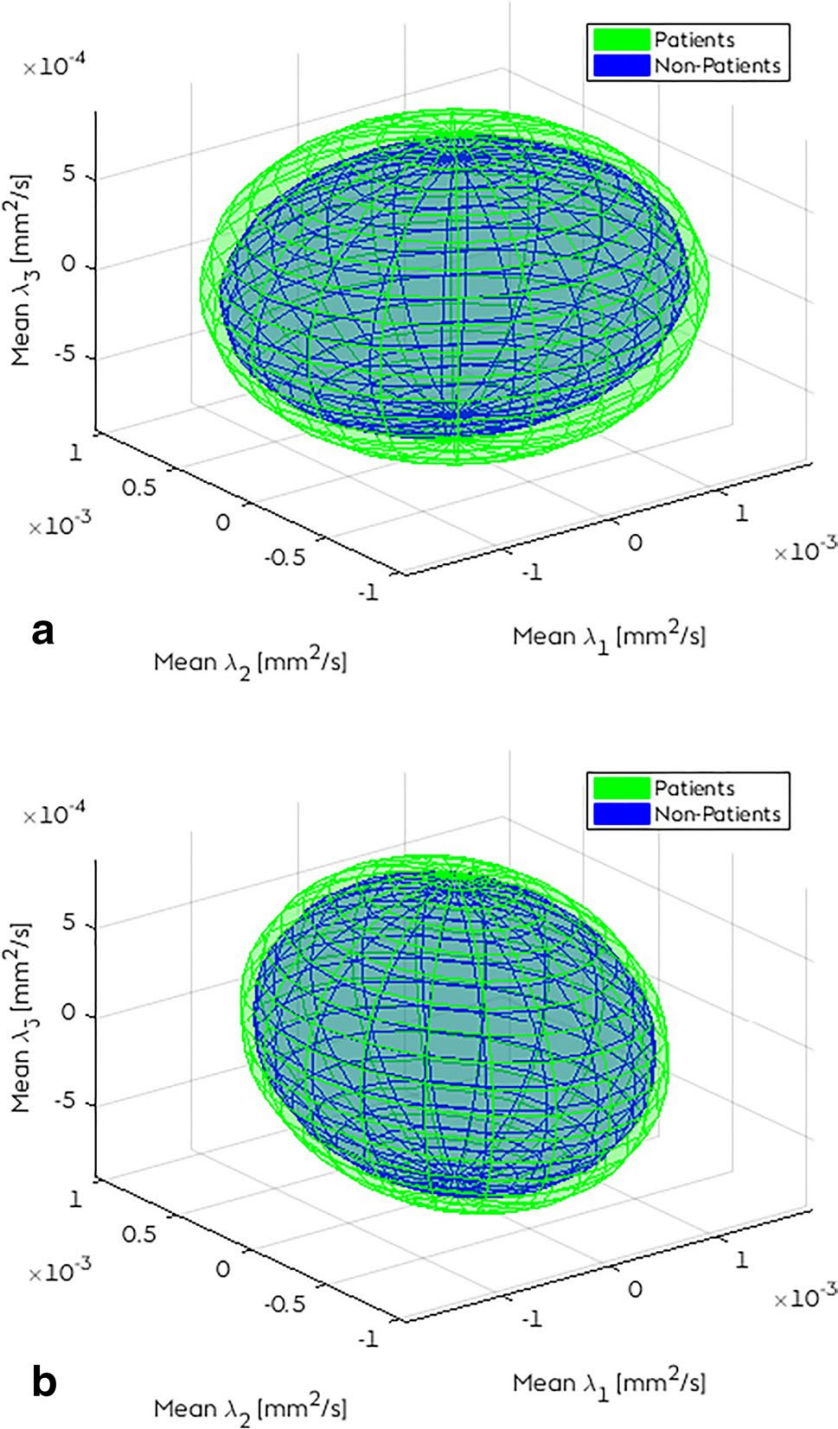
Ellipsoids of the mean *ʎ*1, *ʎ*2, and *ʎ*3 in two tracts: **a** corpus callosum genu and **b** cingulate bundle hippocampal left

The summary shape of the tensors of each voxel in a 3D ellipsoid is shown in Fig. 2 with the mean *ʎ*_1_, *ʎ*_2_, and *ʎ*_3_ of patients and controls for the corpus callosum genu and hippocampal segment of the left cingulate bundle. We see the three major, medium, and minor axis of the diffusion displacement. Both two ellipsoids show any degree of anisotropy or orientation in 3D space. The control group shows smaller ellipsoids in comparison with sCS patients. The corpus callosum shows a more anisotropic ellipsoid than the cingulate bundle, which has a more Gaussian appearance.

### Radial diffusivity analyses

Since the genu of the corpus callosum and the hippocampal segment of the left cingulum bundle show a *ƞ*^2^ > 0.14 in *ʎ*_2_ and *ʎ*_3_, subsequent analyses focused on RD (see Table 3).

**Table 3:** Linear regression on RD with independent variables sCS, gender, age, tract volume, and FOHR (Bonferroni 0.05/2 = 0.025)

	Estimate*	SE*	2.5% CI*	97.5% CI*	*P*-value < 0.025
RD corpus callosum genu					
Intercept	0.58	0.12	0.34	0.83	< 0.025
Syndromic craniosynostosis	0.02	0.05	− 0.07	0.12	0.60
Gender(female)	− 0.01	0.03	− 0.08	0.05	0.67
Age in years	− 0.15	0.04	− 0.24	− 0.07	< 0.025
Tractvolume in mm^3^	0.00	0.00	0.00	0.00	0.10
FOHR per 0.10	0.15	0.03	0.10	0.20	< 0.025
RD hippocampal segment of the left cingulate bundle					
Intercept	0.71	0.08	0.55	0.87	< 0.025
Syndromic craniosynostosis	0.01	0.03	− 0.05	0.06	0.78
Gender(female)	0.01	0.02	− 0.04	0.05	0.80
Age in years	− 0.09	0.03	− 0.14	− 0.04	< 0.025
Tractvolume in mm^3^	0.00	0.00	0.00	0.00	0.52
FOHR per 0.10	0.07	0.02	0.03	0.10	< 0.025

^*^All values are × 10^ − 3

*RD*, radial diffusivity; *FOHR*, frontal occipital horn ratio

By linear regression, we found no significant effect of having syndromic craniosynostosis on RD in the corpus callosum genu and the hippocampal segment of the left cingulate bundle (*p* = 0.60 and *p* = 0.78). The effect of age was significant for both tracts (*p* < 0.025). A rise of 0.1 FOHR gives a rise of 0.15 × 10^−3^ mm^2^/s in RD for the corpus callosum genu (95% CI 0.1 × 10^−3^–0.2 × 10^−3^ mm^2^/s, *p* < 0.025) and a rise of 0.07 × 10^−3^ mm^2^/s for the hippocampal segment of the left cingulate bundle (95% CI 0.03 × 10^−3^–0.10 × 10^−3^ mm^2^/s, *p* = 0.000). The effect of gender and the effect of tract volume were not significant on the RD values in the two assessed tracts.

In Supplemental Tables 1 and 2, subsequent linear regressions based on type of syndrome are depicted. None of the specific syndromes in both linear regressions, on RD of the corpus callosum genu and on RD of the hippocampal segment of the left cingulate bundle, showed to be significantly different compared to the control group.

## Discussion

In this study of white matter microstructure using DTI, we have focused on significant differences of *ʎ*_1_, *ʎ*_2_, and *ʎ*_3_ in the major white matter tracts of 0–2 years old non-operated craniosynostosis patients and controls in the major white matter tracts. Consistent with previous studies of white matter asymmetry [22], our results show lateralization in lambdas. Syndromic craniosynostosis shows not to be a significant factor influencing the DTI parameters in the assessed major white matter tracts. Age and FOHR shows to be significant factors affecting the RD in the tracts corpus callosum genu and the hippocampal segment of the left cingulate bundle.

### Biological effect on increased RD

The corpus callosum is an early myelinated region of the brain, undergoing development in weeks 12 to 16 of pregnancy [23]. During normal brain development and white matter maturation, FA increases and diffusivity (MD, AD, and RD) decreases [24, 25]. Although differences in DTI can demonstrate differences in microstructure, the physics of the measurement is nonspecific and could reflect a variety of mechanisms [22]. As water movement is more restricted perpendicular to myelin membranes than it is parallel to these membranes, it is presumed that RD reflects myelin integrity. Furthermore, RD is determined by axon density and/or diameter of the white matter tract [26, 27].

Our previous study of 7 to 15 years old with sCS, compared to age-matched controls, found increased RD values in the corpus callosum and cingulate bundle [14]. Could this be an intrinsic cause? The fibroblast growth factor receptors have a role in the myelination of the corpus callosum and cingulate gyrus. Wilke et al. showed that in craniofacial development, *FGFR2* and *3* are involved in telencephalon development from which the cingulate bundle and corpus callosum arises [28]. *FGFR* genes are critical to cerebral cortex developmental processes including neuronal migration and stabilization of dendritic patterning [7, 29]. Mutations in the *FGFR* gene could potentially result in abnormal dendritic arborization patterns, measured as increased diffusivity values. *TWIST1* is a transcription factor that is involved in mesodermal differentiation and development [30]. In the current study, we did not find increased diffusivity values in the major white matter tracts in the groups: sCS patients vs controls. Although we did additional analyses comparing specific syndromes to controls (Supplemental Tables 1 and 2), we were not able to find any statistically significant difference compared to controls. However, we do not have enough statistical power to assess this question.

### Mechanical effect on increased RD

We also used the current study to examine for any potential association between brain white matter microarchitecture changes and ventriculomegaly [31, 32]. We used FOHR as a measure of ventriculomegaly and found that in non-operated sCS patients, compared with controls, there was a significant interaction between RD and FOHR in sCS in the corpus callosum genu and the hippocampal segment of the cingulate bundle. In this study, 0.1 increase in FOHR gives a significant increase of RD. Higher RD values could indicate less defined tissue organization, axonal pathology, and reduced myelination or myelin damage [26, 27]. This finding could be related to the mechanical effect of ventriculomegaly. However, it remains unknown if this increase of RD is reversible, if this increase of RD influences cognitive outcome, and, if so, which FOHR cut-off point is optimal for performing a third ventriculostomy or shunt insertion to improve the outcome.

### Limitations

Our study has several limitations. To date, there are no normal ranges of DTI measurements in children under the age of 2 years in literature. DTI is dependent on many technical variables, such as the type of MRI scanner used and the amount of diffusion encoding directions, which makes it extremely difficult to compare absolute DTI values with other DTI studies. Our diffusion protocol may have been overly sensitive. Our use of a 0.1 threshold made it possible to track all structures in the control group and almost all structures in the craniosynostosis group. However, the 0.1 FA threshold meant that more aberrant tracts were generated and additional AND and NOT ROIs were required to exclude aberrant fibers. Though, equal measurements were made between the two groups. Also, the sample size was small and, therefore, we may have failed to identify associations when in fact they do exist, and vice versa.

Analyzing different syndromes of craniosynostosis as one group will bias the outcome. The spectrum of *FGFR* 2 mutations is widely spread, e.g. cognitive functioning of patients with Apert syndrome is significantly different compared to patients with Crouzon syndrome.^1^ As syndromic craniosynostosis differs substantially from each other, it should preferably be analyzed per syndrome. However, we did not have the statistical power to interpret potential differences of the individual syndrome in comparison to controls, as undertaken in the supplemental tables. That said, the current report is the largest DTI study to date in non-operated craniosynostosis patients.

## Conclusion

Before any surgery, microstructural parameters of white matter tracts of syndromic craniosynostosis patients are comparable to those of controls aged 0–2 years. Enlargement of the ventricles plays a significant role in RD in the corpus callosum genu and the hippocampal segment of the cingulate bundle.

## Supplementary Information

Below is the link to the electronic supplementary material.

Supplementary file1 (DOCX 56 KB)

## Abbreviations

AD: Axial diffusivity
FA: Fractional anisotropy
FGFR: Fibroblast growth factor receptors
FOHR: Frontal occipital horn ratio
MD: Mean diffusivity
RD: Radial diffusivity
sCS: Syndromic craniosynostosis
