# Supplementary material for: A diffusion tensor imaging analysis of white matter microstructures in non-operated craniosynostosis patients
Source: Neuroradiology. 2022 Jun 27;64(12):2391–8. doi: 10.1007/s00234-022-02997-8 (PMC9643264; doi:10.1007/s00234-022-02997-8)
Supplement: Supplementary file 1 — Supplementary file1 (DOCX 56 KB) [file 234_2022_2997_MOESM1_ESM.docx]

**Supplemental**

**Supplemental Table 1.** Linear regression on RD Corpus Callosum Genu with independent variables type of syndrome, gender, age, tractvolume and FOHR.

|  | | | | |  |  |
| --- | --- | --- | --- | --- | --- | --- |
|  | **Estimate*** | **SE*** | **2.5% CI*** | **97.5% CI*** | **P value** | |
| (Intercept) | 0.72 | 0.15 | 0.41 | 1.02 | 0.000 |  |
| Apert | 0.05 | 0.07 | -0.09 | 0.20 | 0.484 |  |
| Crouzon | 0.06 | 0.05 | -0.04 | 0.17 | 0.233 |  |
| Muenke | -0.03 | 0.07 | -0.17 | 0.10 | 0.609 |  |
| Saethre-Chotzen | -0.03 | 0.06 | -0.15 | 0.10 | 0.668 |  |
| Complex | 0.02 | 0.06 | -0.11 | 0.14 | 0.806 |  |
| Gender(female) | -0.01 | 0.03 | -0.08 | 0.06 | 0.692 |  |
| Age in years | -0.16 | 0.04 | -0.25 | -0.07 | 0.001 |  |
| Tractvolume in mm3 | 0 | 0 | 0 | 0 | 0.093 |  |
| FOHR per 0.10 | 0.12 | 0.03 | 0.06 | 0.19 | 0.001 |  |

*all values are x 10^-3

**Supplemental Table 2.** Linear regression on RD of the hippocampal segment of the left cingulate bundle, with independent variables type of syndrome, gender, age, tractvolume and FOHR.

|  | **Estimate*** | **SE*** | **2.5% CI*** | **97.5% CI*** | **P value** |
| --- | --- | --- | --- | --- | --- |
| (Intercept) | 0.79 | 0.09 | 0.61 | 0.97 | 0.000 |
| Apert | 0.00 | 0.04 | -0.09 | 0.09 | 0.975 |
| Crouzon | 0.02 | 0.03 | -0.04 | 0.09 | 0.477 |
| Muenke | 0.05 | 0.04 | -0.03 | 0.13 | 0.188 |
| Saethre-Chotzen | -0.01 | 0.04 | -0.08 | 0.06 | 0.779 |
| Complex | 0.01 | 0.04 | -0.07 | 0.08 | 0.876 |
| Gender(female) | 0.00 | 0.02 | -0.04 | 0.05 | 0.948 |
| Age in years | -0.09 | 0.03 | -0.14 | -0.03 | 0.002 |
| Tractvolume in mm3 | 0.00 | 0.00 | 0.00 | 0.00 | 0.597 |
| FOHR per 0.10 | 0.06 | 0.02 | 0.02 | 0.11 | 0.002 |

*all values are x 10^-3

**Abbreviations**: RD: Radial Diffusivity, FOHR: Frontal Occipital Horn Ratio
